# COVID-19 Pandemic Lessons for Creating Effective Mental Health Safety Nets in Lower Middle-Income Countries

**DOI:** 10.7759/cureus.45980

**Published:** 2023-09-26

**Authors:** Osama Bin Khalid, Mustafa Qazi, Almas F Khattak, Madiha Khattak, Muhammad Noman K Wazir, Humaira Gilani

**Affiliations:** 1 Medicine and Surgery, Northwest School of Medicine, Peshawar, PAK; 2 Medicine and Surgery, Northwest General Hospital and Research Center, Peshawar, PAK; 3 Community Medicine and Research, Northwest School of Medicine, Peshawar, PAK; 4 Physiology, Khyber Medical College, Peshawar, PAK; 5 Psychiatry, Northwest General Hospital and Research Center, Peshawar, PAK; 6 Dermatology, Northwest General Hospital and Research Center, Peshawar, PAK

**Keywords:** sustainable solutions, reforms, health system, pandemic, integrated, global health, safety nets, lmics, covid-19, mental health

## Abstract

The coronavirus disease 2019 (COVID-19) pandemic has posed unprecedented challenges to mental health globally, particularly in low- and middle-income countries (LMICs) such as Pakistan. This narrative review aims to synthesize the literature on the impact of the pandemic on mental health in LMICs, the challenges and opportunities for mental health system reform, and the role of safety nets in promoting mental health. A comprehensive search was conducted in several electronic databases, resulting in 35 articles being included for review. Data were extracted and analyzed to identify key themes and trends. The COVID-19 pandemic has led to a significant increase in the prevalence of mental health problems in LMICs, particularly anxiety and depression. This burden is disproportionately borne by vulnerable populations, including women, front-line workers, and those living in poverty. The pandemic has highlighted pre-existing weaknesses in mental health systems in LMICs, including inadequate funding, lack of trained mental health professionals, and stigmatization of mental illness. However, it has also presented opportunities for reform, such as increased awareness and political will, and the use of technology to expand access to mental health services. Building effective safety nets, including social protection programs and community-based interventions, can promote mental health and address social determinants of mental illness. The COVID-19 pandemic has underscored the urgent need for mental health system reform and the development of effective safety nets in LMICs. Policymakers should prioritize investment in mental health and address the social determinants of mental illness to build more resilient societies.

## Introduction and background

Background and context

Global mental health has been significantly impacted by the coronavirus disease 2019 (COVID-19) pandemic, particularly in low- and middle-income (LMIC) nations like Pakistan [1]. Building efficient safety nets for mental health is therefore more important than ever. The pandemic has increased the frequency of mental health issues worldwide, notably in LMICs [1], and has brought attention to the need for adequate and ready mental health services to lessen the harmful effects of upcoming crises. The pandemic's impact on mental health is not limited to those who have contracted the virus, but also on the general population, who have been affected by the economic downturn and the stringent containment measures imposed by the government. In Pakistan, the pandemic disturbed daily life and contributed to a rise in anxiety, depression, and other mental health issues [2].

The World Health Organization (WHO) recognized the importance of mental health services during the pandemic and has called for an urgent scale-up of mental health services [3]. LMICs frequently have inadequate resources and weak mental health systems, making them especially vulnerable. In Pakistan, for example, the epidemic aggravated pre-existing mental health issues while creating new ones, resulting in an increase in the prevalence of mental health issues such as anxiety and depression [4,5]. The mental health system in Pakistan was already under-resourced and fragmented, which made it even more challenging to address the increased demand for mental health services during the pandemic.

The pandemic has also underlined the importance of adequate mental health safety nets in LMICs. Safety nets are social protection systems that help individuals and communities prevent and alleviate the negative effects of shocks and stresses, including mental health crises [6]. Despite the necessity of safety nets, many LMICs lack effective mental health policies and initiatives, leaving their citizens more susceptible during the pandemic. As a result, there is an urgent need to understand how to construct effective mental health safety nets in LMICs, with a focus on lessons acquired from the COVID-19 pandemic in Pakistan.

Objective

This review aims to thoroughly assess the effects of the COVID-19 pandemic on the mental health landscape throughout Pakistan, with a focus on current mental health conditions and their incidence rates. Furthermore, it aims to identify the underlying drivers responsible for the rise in mental health issues during the epidemic in Pakistan. Additionally, the review will examine the effectiveness of current mental health initiatives and legislation in Pakistan in tackling the growing mental health concerns brought to the forefront by the pandemic. Finally, this review intends to offer a series of solutions for the construction of strong mental health safety nets within Pakistan, using significant insights from the COVID-19 pandemic's experiences and lessons, including the role of technology. The findings from this study can inform policy and practice for building effective safety nets for mental health in LMICs.

Research questions

The COVID-19 pandemic's influence on mental health in Pakistan, as well as variations in the prevalence of prevalent mental health issues since its inception, raise serious questions. These include an investigation into the contextual elements that contributed to heightened mental health concerns amid the pandemic, such as the harshness of lockdown measures and the virus itself. Furthermore, evaluating Pakistan's existing mental health policies and initiatives, as well as their effectiveness in tackling pandemic-related concerns, is critical.

To develop effective mental health safety nets in Pakistan, it is critical to evaluate diverse solutions while drawing lessons from the COVID-19 pandemic. This entails assessing the strengths and shortcomings of present mental health programs and putting in place evidence-based measures that not only address urgent needs but also promote long-term resilience in the face of future health crises.

Methodology

Search Strategy

We conducted a comprehensive literature search using several databases, including PubMed, Medline, PsychINFO, and Google Scholar. The search terms used included "COVID-19," "pandemic," "mental health," "LMICs," and "Pakistan." We also reviewed the reference lists of relevant articles to identify additional studies. The search and screening process was conducted by two independent reviewers. Any disagreements were resolved through discussion and consensus. The final set of studies included in the narrative review was determined based on relevance to the research questions and study objectives.

Inclusion Criteria and Exclusion Criteria

Articles had to meet the following criteria to be included: (i) published in English, (ii) focus on mental health during the COVID-19 pandemic in Pakistan, (iii) include quantitative or qualitative data on mental health outcomes, policies, or programs, and (iv) published between January 2020 and 2023. Studies were excluded if they were published in a language other than English, focused on mental health issues in countries other than Pakistan, lacked original data on the consequences of mental health policies, or initiatives, or were published before January 2020.

Data Extraction and Analysis

Data extraction was conducted by two independent reviewers using a standardized data extraction form. The following information was extracted from each study: author(s), year of publication, study design, study population, sample size, mental health outcome measures, mental health policies and programs, and key findings related to the impact of the COVID-19 pandemic on mental health in Pakistan. Any discrepancies in data extraction were resolved through discussion and consensus. A narrative synthesis approach was used to analyze the data. The extracted data were grouped into themes based on the research questions and study objectives. The themes were analyzed using a thematic analysis approach, which involved identifying patterns and trends in the data and interpreting the findings in relation to the study objectives. The analysis included a description of the mental health outcomes, policies, and programs identified in the studies, as well as an evaluation of the strengths and weaknesses of these policies and programs. Finally, the analysis identified strategies for building effective safety nets for mental health in LMICs, with a focus on Pakistan.

The narrative synthesis approach was chosen because it allows for the integration of data from different study designs and methodologies, which is important given the limited research on mental health during the COVID-19 pandemic in LMICs, including Pakistan. The approach also enables the identification of key themes and trends and the development of recommendations for future research and policy initiatives.

Quality Assessment

The quality of the studies included in the narrative review was assessed using the Mixed Methods Appraisal Tool (MMAT) version 2018. The MMAT is a comprehensive tool for evaluating the quality of studies that use quantitative, qualitative, and mixed-methods research designs. It includes five categories of criteria, each with a set of specific questions that are used to assess the quality of the studies. Two independent reviewers conducted the quality assessment using the MMAT. Any discrepancies in quality assessment were resolved through discussion and consensus. The studies were rated as low, moderate, or high quality based on the MMAT criteria. The quality assessment ensured that only high-quality studies were included in the narrative review. This helped to minimize the risk of bias and to ensure that the findings were robust and reliable. The results of the quality assessment were also used to identify areas for future research and to develop recommendations for policy and practice.

## Review

Overview of the mental health burden in LMICs

With an estimated 10-20% of the world's population suffering from mental health illnesses, mental health represents a serious global health challenge [7]. Due to the estimated higher frequency of mental health issues in LMICs than in high-income countries, this burden is particularly severe in these nations [8]. Due to a lack of access to mental healthcare and the stigma surrounding mental illness, mental health issues are frequently underdiagnosed and undertreated in LMICs [8]. With detrimental effects on health, education, employment, and economic production, this places a heavy cost on people, families, and communities [7]. Despite the enormous burden of mental illness in LMICs, these nations lack funding for mental health research and resources [8]. This underlines the requirement for increased funding for mental health services, laws, and initiatives that are specially designed to meet the requirements and conditions of LMICs.

The COVID-19 pandemic's effects on mental health in LMICs

People's mental health has been significantly impacted by the COVID-19 pandemic all throughout the world, notably in LMICs [9]. Numerous LMICs are seeing higher rates of anxiety, depression, and other mental health illnesses as a result of the pandemic and its related containment measures, such as lockdowns and physical segregation [10]. In Pakistan, for example, there has been a significant increase in the prevalence of anxiety and depression during the pandemic, with many people attributing this to financial strain, social isolation, and future uncertainty [11]. The pandemic has brought attention to the nation's pre-existing mental health issues, such as the stigma attached to mental illness and restricted access to mental health services [11]. Similar patterns have been seen in other LMICs, including India, where the epidemic has increased the incidence of post-traumatic stress disorder (PTSD), anxiety, and depression [12].

The COVID-19 pandemic's effects on mental health in LMICs are intricate and multifaceted, influenced by a variety of variables, including stressors at the individual and community levels, socioeconomic variables, and access to mental health services [9]. Addressing the mental health impacts of the pandemic in LMICs will require a multi-sectoral approach, involving investment in mental health services and policies, as well as addressing broader social and economic determinants of mental health [10].

Prevalence and impact of mental health problems in Pakistan

The COVID-19 pandemic has had a significant impact on the mental health of individuals worldwide, including those in Pakistan. Studies have reported high levels of stress, anxiety, depression, and psychological distress among the Pakistani population due to the pandemic and related restrictions [13,14]. In addition to the direct impact of the virus, the pandemic has also led to economic instability, social isolation, and increased domestic violence, which have further exacerbated mental health issues [15]. According to a Pakistani study, there was a considerable rise in the prevalence of depression and anxiety symptoms throughout the pandemic, with healthcare workers experiencing the greatest rates [16]. In accordance with a different study, the epidemic has increased Pakistan's suicide rates, underscoring the urgent need for mental health interventions [17]. Additionally, the COVID-19 pandemic has brought attention to the inequities in Pakistan's access to mental healthcare. In Pakistan, mental health services are frequently underdeveloped, underfunded, and stigmatized, which makes it challenging for people to access the right care [18].

Existing mental health systems in LMICs

Mental health systems in LMICs face numerous challenges, including limited funding, inadequate human resources, and weak governance structures. In many LMICs, mental healthcare is primarily provided through the public sector, with limited resources and infrastructure dedicated to mental health. In some cases, mental health services may be integrated into primary care or provided through non-governmental organizations (NGOs) and community-based programs. However, there are often disparities in access to mental healthcare, with those in rural or marginalized communities having limited or no access to services [19]. The WHO's Mental Health Gap Action Programme (mhGAP), which aims to establish evidence-based guidelines for the management of mental diseases in primary care settings, is one recent attempt to build mental health systems in LMICs [20]. Other programs, like the Pastoralist Areas Resilience Improvement through Market Expansion (PRIME) program in Ethiopia and the Friendship Bench program in Zimbabwe, have prioritized community-based treatments and task-shifting to increase access to mental health care. Despite these initiatives, there is still a sizable treatment gap for mental illnesses in LMICs, with a high percentage of those who need care not receiving it. The introduction of evidence-based therapies suited to the particular requirements of LMICs as well as increased investment in mental health systems are necessary to close this treatment gap.

Challenges and opportunities for mental health system reforms in LMICs

There is a growing recognition of the importance of mental health in LMICs, and several international organizations, including the WHO, have developed strategies to improve mental health services in these countries. The WHO's Mental Health Action Plan 2013-2020 includes a focus on strengthening mental health services in LMICs, as well as improving access to essential psychotropic medications and reducing stigma and discrimination [21]. In Pakistan, the government has also recognized the need to prioritize mental health and has developed a National Mental Health Policy to improve mental health services in the country. The policy includes a focus on integrating mental health into primary healthcare, improving access to essential psychotropic medications, and reducing stigma and discrimination [22].

Limited finance is one of the biggest problems facing mental health services in LMICs. The emphasis assigned to mental health issues is frequently lower than that given to other health issues, which results in underfunding and insufficient resources [8]. In rural areas, in particular, this has resulted in a dearth of mental health facilities and qualified mental health workers. Additionally, it is frequently impossible for those who cannot afford to pay for treatments out of pocket to obtain mental health care because it is not frequently covered by insurance or other healthcare financing methods. 

A challenge facing mental health systems in LMICs is the shortage of trained mental health professionals [20]. According to the WHO, many LMICs have less than one mental health professional per 100,000 population, which is well below the recommended minimum of 10 mental health professionals per 100,000 population. This shortage is particularly acute in rural areas, where access to mental health care is often limited [8]. Social stigma surrounding mental health issues is another significant challenge in LMICs [23]. Mental health problems are often viewed as a sign of weakness or personal failure, which can lead to discrimination and social exclusion. This stigma can prevent individuals from seeking help and can also discourage investment in mental health systems by policymakers and donors. Addressing these challenges will require a concerted effort by governments, international organizations, and civil society to prioritize mental health and invest in strengthening mental health systems in LMICs.

The incorporation of mental health into primary healthcare presents an opportunity for mental health system reform in LMICs. Many persons seeking health services in LMICs start with basic care, and including mental health services in primary care can increase access to mental health care services. Access to mental health services in LMICs has been found to be improved by community-based methods of healthcare delivery [24]. The use of technology to expand access to mental health treatment presents another chance for mental health system reform in LMICs. Delivering mental health care in LMICs has been accomplished with the help of telemedicine and mobile health interventions [25]. These technologies can overcome geographic barriers to access, reduce the stigma associated with seeking mental health services, and increase the efficiency of service delivery. Investing in mental health research is another important opportunity for mental health system reform in LMICs. Research can generate evidence to guide the development and implementation of effective mental health policies and programs [26]. Additionally, research can provide information on the burden of mental health problems in LMICs and identify effective interventions for addressing these problems.

Overall, while mental health systems in LMICs face numerous challenges, there are also opportunities for reform that can be harnessed to improve the delivery of mental health services. The integration of mental health into primary health care, community-based approaches to mental health care delivery, the use of technology to increase access to mental health services, and investment in mental health research are all important opportunities for mental health system reform in LMICs. Furthermore, the pandemic has led to innovative approaches to mental health service delivery, such as telemedicine and online counseling, which could improve access to mental health services in LMICs [27].

Mental health system reform in Pakistan during the pandemic

Pakistan, like many other LMICs, faced numerous challenges in providing effective mental health care to its population, especially during the COVID-19 pandemic [28]. However, the pandemic has also provided an opportunity for mental health system reform in Pakistan.

One of the key initiatives taken by the Pakistani government during the pandemic was the launch of the Corona Stress Counseling Helpline in April 2020. This helpline provided free counseling services to individuals suffering from stress and anxiety due to the pandemic. The helpline was staffed by trained psychologists and mental health professionals who provided counseling in Urdu, the national language of Pakistan. By the end of 2020, the helpline had received over 200,000 calls, indicating a significant demand for mental health services in the country [29].

Another significant development during the pandemic was the introduction of telemedicine services in mental health care. Prior to the pandemic, telemedicine was not widely used in Pakistan for mental health care, but the pandemic led to the rapid expansion of telemedicine services in the country. Telemedicine services were used to provide counseling and therapy sessions to individuals who were unable to visit mental health clinics in person due to lockdowns and other pandemic-related restrictions [30].

In Pakistan, there have been proposals for more extensive reforms to the mental health system in addition to existing measures. The integration of mental health services within the primary healthcare system is a crucial suggestion. Currently, tertiary care facilities, which are located in urban areas, provide the majority of mental health care in Pakistan. As a result, many people living in rural locations lack access to mental health services. Mental health services can be made more widely accessible across the nation by incorporating them into the primary healthcare system.

Building effective safety nets for mental health in LMICs

Safety nets for mental health refer to the support and care structures in place for individuals facing mental health challenges. In LMICs, such safety nets are often inadequate, resulting in limited access to mental health care and services.

The COVID-19 pandemic has highlighted the critical importance of having strong and effective safety nets for mental health in LMICs. There is an urgent need for these countries to invest in their mental health systems and build capacity to address the mental health burden, particularly in the context of the pandemic [8]. There are several strategies that LMICs can use to build effective safety nets for mental health, including scaling up mental health services to ensure that they are available and accessible to those who need them. This includes investing in community-based mental health services and integrating mental health into primary care. The mental health workforce needs to be strengthened as there is a shortage of mental health professionals in LMICs, and this needs to be addressed through increased investment in training and capacity building [31,32]. This will ensure that there are enough mental health professionals to provide services and support to those in need. The community should be engaged and their participation participation should be encouraged. Empowering communities to take an active role in mental health promotion and prevention can help reduce stigma and increase access to services [33]. The latest available technology should be leveraged; technology can be a powerful tool for improving mental health care in LMICs. Telemedicine and mobile health interventions can help increase access to care, particularly in hard-to-reach areas. Furthermore, LMICs need to address the social determinants of mental health, including poverty, unemployment, and discrimination. This will require a multisectoral approach that involves collaboration between mental health professionals, policymakers, and other stakeholders [34]. Investing in these strategies can help build effective safety nets for mental health in LMICs and ensure that these countries are better prepared for future global health crises.

The WHO has emphasized the importance of establishing community-based mental health services as a key strategy for strengthening mental health systems in LMICs. Such services can include community outreach programs, peer support groups, and community mental health centers. These services can be especially effective in rural areas where access to mental health services may be limited. Additionally, telemedicine and mobile health technologies, which have emerged as potential tools, can provide remote consultation, counseling, and medication management. Research has shown promising results for telemedicine-based mental health interventions in LMICs, particularly in reducing symptoms of depression and anxiety [35,36].

Recently, the Pakistan government launched Humraaz, a mental health application, along with an integrated helpline of 1166, to address the prevalent issue of poor mental health and lack of access to treatment in the country [37]. The initiative was developed in collaboration with the Ministry of Health Services, Regulations and Coordination, WHO, the National Information Technology Board, and the Federal Directorate of Immunization. The app provides a one-stop solution for citizens with depression, self-harming thoughts, and other mental health issues. It includes features such as guided counseling, psychotherapy sessions, daily activities monitoring, 24/7 connection with professionals, an international standard knowledge base, and WhatsApp support. The initiative has more than 140 registered psychologists/psychiatrists, out of which more than 60 are psychiatry resident doctors and counselors. Moreover, the app has support groups of people for help and sharing experiences. The toll-free helpline 1166 is also active, and more than 30 government psychologists/psychiatrists are on board to provide free consultation services to citizens with mental health concerns and self-harming thoughts. The Humraaz app and helpline are essential components of the safety nets for mental health in Pakistan, which have been expanded in recent years to address the challenges faced by people with mental health problems. The initiative provides a promising opportunity for Pakistan to improve access to mental health services, reduce stigma around mental health, and promote mental well-being.

Furthermore, the integration of mental health services into primary care systems has been identified as a promising approach to increasing access to mental health care in LMICs. This approach can address the shortage of mental health professionals and reduce the stigma associated with seeking mental health care. The WHO has recommended the integration of mental health into primary care systems as a cost-effective and feasible strategy for improving mental health care in LMICs [38].

Recommendations

Based on the current review, several recommendations can be made for building effective safety nets for mental health in LMICs. First, there is an urgent need to raise mental health awareness and literacy. This entails increasing public awareness of mental health concerns and educating the public on the need to seek help when necessary. Community-based interventions and robust public health campaigns can help attain such goals. Second, increased mental health spending in LMICs is critical. This can be accomplished by combining domestic and foreign financial sources, as well as novel financing vehicles such as social impact bonds. Third, there is a pressing necessity to reinforce the mental health workforce in LMICs. This includes expanding the number of qualified mental health experts and improving working conditions for them. This goal can be met by implementing targeted training programs, improving compensation packages, and creating more supportive work cultures. Fourth, increasing access to mental health care is critical. Task-shifting, the use of technology such as telemedicine, and the integration of mental health services into primary care are all ways that can be used to increase access to these services. Fifth, it is critical to address the social determinants of mental health in LMICs. This includes addressing challenges such as poverty, unemployment, and social marginalization. To accomplish this, a combination of social protection programs, job creation initiatives, and community-based interventions should be considered. Finally, improving mental health governance is critical. This includes strengthening policy and regulatory frameworks as well as enhancing stakeholder participation. The development of national mental health policies and the establishment of multi-stakeholder mental health governance platforms are critical steps in achieving this goal.

Implementing these recommendations will require concerted efforts by governments, civil society organizations, international organizations, and other stakeholders. However, the benefits of investing in mental health in LMICs are clear, not only in terms of improving the health and well-being of populations but also in terms of promoting economic development and social inclusion.

Role of Technology 

The role of technology in expanding access to mental health services has gained significant attention in recent years, especially in LMICs where access to mental health services is limited. The COVID-19 pandemic has highlighted the need for innovative and cost-effective ways to deliver mental health services remotely.

One of the most promising technologies for expanding access to mental health services in LMICs is mobile health (mHealth). mHealth interventions use mobile devices such as smartphones and tablets to deliver mental health interventions remotely. These interventions can include text messaging-based interventions, mobile apps, and video-based teletherapy. Several studies have shown the potential of mHealth interventions in improving mental health outcomes in LMICs. A randomized controlled trial conducted in Pakistan found that a text messaging-based intervention was effective in reducing symptoms of depression and anxiety among primary care patients with comorbid physical health conditions [39]. Similarly, a systematic review of mHealth interventions for mental health in LMICs found that these interventions were effective in improving a range of mental health outcomes, including depression, anxiety, and post-traumatic stress disorder [40].

Another technology that holds promise for expanding access to mental health services in LMICs is telemedicine. Telemedicine involves using videoconferencing technology to provide mental health services remotely. Telemedicine can be particularly effective in reaching individuals living in remote and underserved areas, where access to mental health services is limited.

While technology has the potential to expand access to mental health services in LMICs, there are several challenges that need to be addressed. One of the main challenges is the lack of infrastructure and resources to support these interventions. Additionally, there are concerns about the privacy and security of personal health information in the context of mHealth and telemedicine interventions

Implications for policy and practice

The findings of this narrative review have several implications for policy and practice in LMICs, particularly in Pakistan. First, there is a need to prioritize mental health in policy and practice. This includes developing and implementing national mental health policies, increasing funding for mental health services, and promoting awareness and education about mental health. Second, mental health systems in LMICs should be reformed to address the challenges identified in this review, such as lack of resources, insufficient workforce, and limited access to care. This may involve developing new models of care that are community-based, culturally sensitive, and integrated with other health services. Third, technology can play a crucial role in expanding access to mental health services in LMICs, particularly in remote or underserved areas. This may include the use of telemedicine, mHealth applications, and other digital technologies. Finally, building effective safety nets for mental health requires a comprehensive approach that addresses the social determinants of mental health, such as poverty, social exclusion, and violence. This may involve collaboration between the health sector and other sectors, such as education, employment, and social welfare.

Limitations and future directions for research

Despite the valuable contributions of the studies reviewed, there are several limitations that should be acknowledged. First, the majority of studies included in this review were cross-sectional and relied on self-reported data, which may have limitations in terms of accuracy and reliability. Longitudinal studies with objective measures would provide a more robust understanding of the impact of the pandemic on mental health in LMICs. Second, the studies included in this review focused primarily on the immediate impact of the pandemic on mental health and did not address the potential long-term effects.

In terms of future directions for research, there is a need for more studies that examine the impact of the pandemic on specific vulnerable populations in LMICs, such as women, children, and individuals with pre-existing mental health conditions. Additionally, there is a need for research that examines the effectiveness of specific interventions and safety nets for mental health in LMICs, as well as the barriers to their implementation. Finally, more research is needed to examine the role of technology in expanding access to mental health services in LMICs, as this has the potential to address some of the challenges faced by traditional mental health systems in these settings.

## Conclusions

Mental health is an important aspect of overall well-being, yet it is often overlooked and neglected in LMICs due to several challenges. The COVID-19 pandemic has had a significant impact on mental health globally, particularly in LMICs like Pakistan, where there were already significant mental health challenges. The pandemic has exacerbated these challenges and highlighted the need for effective safety nets for mental health in LMICs. There is a need for a comprehensive approach to mental health system reform in LMICs, including strengthening primary care services, integrating mental health into general health services, and increasing investment in mental health research. Additionally, there is a need for a robust and sustainable funding model for mental health services in LMICs. Technology has the potential to play a critical role in expanding access to mental health services in LMICs. Telemedicine and digital mental health interventions can help to bridge the gap between mental health services and those who need them, particularly in remote and underserved areas. Overall, the review underscores the urgent need for building effective safety nets for mental health in LMICs and recommends a holistic approach to mental health system reform that addresses the challenges and leverages opportunities for change.

Pakistan's mental health system reform during the pandemic highlights the potential of integrating technology and involving multiple stakeholders in addressing mental health challenges. The launch of Humraaz, a mental health application, and an integrated helpline in Pakistan is a promising step towards addressing the mental health burden in the country. Overall, policymakers and practitioners in LMICs need to prioritize mental health and work towards creating a comprehensive and effective mental health system that can provide support and treatment to those in need.
